# Similar and Different Gray Matter Deficits in Schizophrenia Patients and Their Unaffected Biological Relatives

**DOI:** 10.3389/fpsyt.2013.00150

**Published:** 2013-11-21

**Authors:** Yuan Xiao, Wenjing Zhang, Su Lui, Li Yao, Qiyong Gong

**Affiliations:** ^1^Department of Radiology, Huaxi MR Research Center (HMRRC), West China Hospital, Sichuan University, Chengdu, China

**Keywords:** schizophrenia, unaffected relatives, MRI, voxel-based morphometry, gray matter, meta-analysis

## Abstract

Neuroimaging studies have revealed significant reductions in the gray matter (GM) of several brain regions in patients with schizophrenia, a neuropsychiatric disorder with high hereditability. However, it is unclear whether unaffected relatives have GM abnormalities in common with their affected relatives, which may relate to susceptibility to developing schizophrenia. To address this issue, we conducted two separate meta-analyses of voxel-based morphometry to investigate GM abnormalities in schizophrenia patients and their unaffected relatives. One meta-analysis compared a patient group with healthy controls, whereas the other meta-analysis compared the unaffected relatives with healthy controls. Eight studies comprising 495 patients with schizophrenia, 584 unaffected relatives of patients, and 596 healthy controls were systematically included in the present study. Compared to healthy controls, the patient group showed decreased GM in the right cuneus, the right superior frontal gyrus, the right insula and the left *claustrum*, and increased GM in the bilateral *putamen*, the right parahippocampal gyrus, the left precentral gyrus, the left inferior temporal gyri, and the right cerebellar tonsil. The comparison between unaffected relatives and healthy controls showed a GM reduction in the left *claustrum*, the bilateral parahippocampal gyri, the left fusiform gyrus, the right inferior temporal gyrus, and the bilateral medial prefrontal cortices, whereas increased GM was observed in the right hippocampus, the right fusiform gyrus, the right precentral gyrus, and the right precuneus. Thus, our meta-analyses show that the GM changes in schizophrenia patients and their unaffected relatives are largely different, although there is subtle overlap in some regions.

## Introduction

Anatomical brain abnormalities have been well established in schizophrenia (1, 2). However, the molecular mechanism that underlies anatomical brain abnormalities in schizophrenia remains unclear, and an important question is whether these abnormalities are related to the illness itself or are linked to genetic risk, as an endophenotype (3). Characterizing endophenotypes is a compelling venture because endophenotypes may represent less complex disease antecedents that are easier to study than the illness syndrome itself. As many brain structures are highly heritable (4) and are influenced by genetic variants that are beginning to emerge in large-scale studies (5), brain structures may be a good endophenotype (3). Previous studies of unaffected relatives of schizophrenia patients demonstrated gray matter (GM) abnormalities that were similar but less pronounced than those in the patients, suggesting that the abnormalities are related to genetic susceptibility to the disorder (6, 7).

The majority of previous morphometric studies, however, studied schizophrenia patients and their unaffected relatives separately. Decreased GM volume in the left superior temporal gyrus and the left medial temporal lobe were the most common findings in the comparative studies of patients with schizophrenia and healthy controls with no personal or family history of psychosis (2). However, the results observed in such comparisons may reflect a complex combination of multiple causes, such as genetics, substance abuse, environmental factors, obstetric complications and birth injuries (8, 9), and other factors secondary to the illness. Indeed, whether these changes are the result of the use of antipsychotic medication or the severity of the illness itself is a matter of debate. However, studying unaffected relatives avoids the confounding factors of chronicity and antipsychotic medication. Furthermore, this population is independent of psychosis, thus avoiding the possible neurotoxic effects of psychosis, which may be present even in high-risk populations. Finally, a focus on unaffected relatives makes it possible to study genetic factors and environmental factors with respect to their roles in schizophrenia etiology (1). The most commonly reported abnormal brain region in unaffected relatives is the hippocampus, which was confirmed in a meta-analysis of non-psychotic first-degree relatives of schizophrenia patients (10). However, it should be noted that the hippocampus is also one of the most commonly investigated brain regions in studies of unaffected relatives of schizophrenia patients using region-of-interest (ROI) analysis. Only a few studies have employed whole-brain data-driven unbiased methods, such as voxel-based morphometry (VBM), which performs voxel-wise comparisons of gray and white matter probabilities between groups of subjects. The most common findings in whole-brain comparison studies comparing unaffected relatives with healthy controls involve the left putamen/globus pallidus (basal ganglia), the amygdala, and the parahippocampal gyrus (11), which are similar to the results of studies involving patients. However, one important problem with independent studies of patients and unaffected relatives is that the patients and unaffected relatives are not biologically related; thus, one cannot conclude from these studies whether the patients share anatomical deficits with their unaffected relatives. Only a few studies have explored whole-brain anatomical deficits in both patients and their unaffected biological relatives (6, 7, 12–17), but the findings are inconsistent. Hu et al. (17) found that untreated patients with first-episode schizophrenia and their unaffected siblings shared decreases of GM in the left middle temporal gyrus. A study of discordant monozygotic twins found that non-psychotic twins showed no significant differences in regional GM relative to the healthy control twins, although twins with schizophrenia had smaller GM in the insula, superior/medial frontal, pre/postcentral, cingulate, and superior temporal gyri compared to healthy control twins (13). Negative results of GM in first-degree relatives were also found in another study (12), though patients with schizophrenia reported reduced GM primarily in the anterior cingulate gyrus and the insula. In an optimized VBM study, Honea et al. (14) investigated a large group of patients and their unaffected siblings and found that siblings tended to share GM decreases in the medial frontal, superior temporal, and insular cortices with their affected siblings, but these decreases were not significant after multiple comparison correction.

Thus, the aims of the present meta-analysis were to determine whether schizophrenia patients and their unaffected biological relatives have GM abnormalities and if so, to map the common and the different alterations of GM between both groups.

## Materials and Methods

### Selection procedures

Studies were selected using a systematic search strategy. Two independent researchers conducted a two-step literature review process. First, we performed a database search to find putative VBM studies. The keywords included (1) schizophrenia, (2) families or relatives or sibling or parents or offspring or twins, (3) structural MRI, (4) VBM or voxel-based analysis. These items were entered in various combinations when searching. Only articles published in print or online before July 2013 in English were selected using two databases (Pubmed and Medline). Second, the reference lists of the selected articles and similar studies were manually checked for additional studies that fit these criteria but that were not identified by our computerized searching process. We included all studies that met the following criteria: (a) an original paper in a peer-reviewed journal; (b) investigating GM volume or GM density/concentration by VBM at the whole-brain level among schizophrenia patients, their relatives or siblings and healthy controls; and (c) use of Montreal Neurological Institute (MNI) or Talairach coordinates in the VBM analyses. Studies that had overlapping samples were excluded. Studies lacking biological relatives of patients with schizophrenia were also excluded. The relatives investigated in each study were supposed to come from the studied patients’ own families, not from other families with a history of schizophrenia. We contacted the corresponding authors for details required for the present meta-analysis. In our present meta-analysis, Meta-analysis Of Observational Studies in Epidemiology (MOOSE) guidelines are followed (18).

### Voxel-based meta-analysis

Prior to conducting the voxel-based meta-analysis, we extracted peak coordinates resulting from VBM analyses across the whole-brain and then excluded those coordinates using partial coverage, ROIs, or small volume correction (SVCs). This approach ensured that the studies we collected were comparable and that their purpose was to localize GM alterations across the entire cortex. Within each included study, the same statistical threshold used throughout the whole-brain was carefully checked to avoid biases toward liberally thresholded brain regions, as it is not uncommon in neuroimaging studies that the statistical thresholds for some ROI are more liberal than for the rest of the brain (19).

The Effect-Size Signed Differential Mapping (ES-SDM) (http://www.sdmproject.com/software), which is a new version of SDM, was chosen as our primary meta-analytical software (20). ES-SDM has several advantages over other voxel-based methods. First, the criterion that ES-SDM uses for selecting reported peak coordinates is so strict that it ensures that only regions with statistical significance at the whole-brain level will be considered in the meta-analysis, which prevents biases resulting from methods using liberal thresholds and ROI methods in neuroimaging studies (19). Second, previous methods such as activation likelihood estimation (ALE) (21) and multilevel kernel density analysis (MKDA) (22) only need reported peak coordinates. As for the ES-SDM, the use of effect-size enables combination of reported peak coordinates with statistical parametric maps allowing more detailed and accurate meta-analysis (20). Third, to avoid any voxel appearing significant in opposite directions, ES-SDM permits the representation of both GM increases and decreases in the same map. Last, complementary analyses such as jack-knife, subgroup, and meta-regression analyses are readily used to assess the robustness and heterogeneity of the results (20).

Two meta-analyses were conducted separately to describe differences in GM between patients and healthy controls and between relatives and healthy controls. A Gaussian kernel of 20 mm half-width was employed to assign indicators of proximity to the reported coordinates as it can optimally balance sensitivity and specificity in ES-SDM (20). A systematic voxel-based leave-one-out jack-knife analysis was also conducted to test the replicability of the results in various combinations of the included studies. The findings might be highly conclusive and replicable if previous significant ES-SDM results could be replicated in all or most study combinations. The standard ES-SDM thresholds (uncorrected *P* < 0.005, extent threshold of clusters >10 voxels) which were proposed to optimally balance the sensitivity and specificity and to be an approximate equivalent to corrected *P*-value = 0.05 in ES-SDM for effect-size were used (20).

## Results

### Included studies

**Figure 1:**
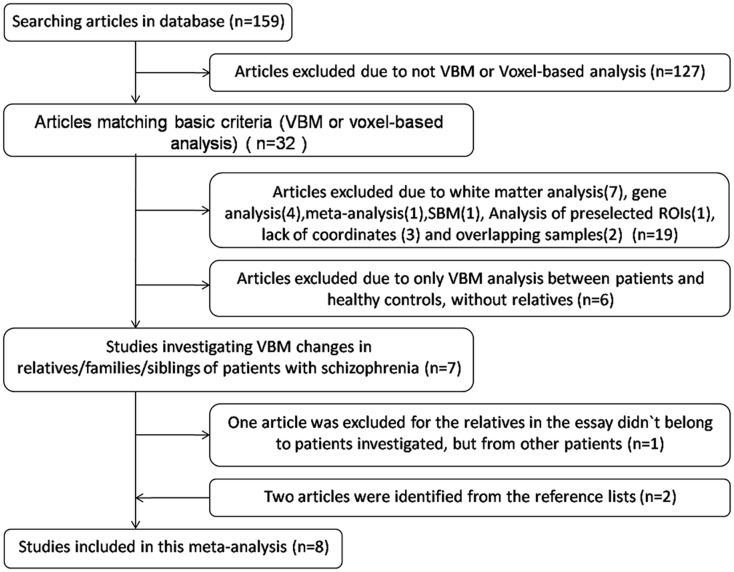
**Flow diagram of inclusion and exclusion criteria for the meta-analysis**.

The search flow diagram is shown in Figure 1. Of the 159 initial hits identified by the database search, 127 articles were excluded for not using VBM or voxel-based analysis, by reading their abstracts. Of the remaining 32 articles, 25 did not meet the predefined inclusion criteria (because of factors such as carrying out only white matter analysis, lack of information or data from relatives of patients with schizophrenia, use of overlapping samples). The other seven articles were shortlisted for using VBM to investigate GM changes in patients, unaffected relatives, and healthy controls. However, further investigation revealed that in one of the seven articles, the relatives investigated were not from the investigated patients’ families, but from other families. The remaining six articles, together with two articles identified from the reference lists, comprised the final list of eight articles to be included. Seven of eight studies included different types of first-degree relatives, namely, one of monozygotic discordant twins (13), three of siblings (12, 14, 17), two of parents (7, 15), one of both parents and siblings (6). Only one study included the first- and second-degree of relatives (16). Among the included articles, four utilized GM density while the other four utilized GM volume to measure differences between groups. A total of 495 patients with schizophrenia, 584 relatives of patients, and 596 healthy controls were included. All of the studies included both types of VBM comparison (i.e., patients vs. controls and relatives vs. controls). In addition, Lui et al. (15) distinguished patients with familial schizophrenia from patients with sporadic illness and no family history. Thus, Lui et al. provided two VBM comparisons for each group, because the two groups of patients and relatives were separately compared with healthy controls. Consequently, each meta-analysis contained nine datasets. The demographic characteristics of the patients, relatives of patients with schizophrenia, and healthy controls are presented in Tables 1 and 2.

**Table 1:** **Demographic characteristics of the studies comparing schizophrenia patients and healthy controls**.

Study	Sample size		Age in years (SD)		Male in proportions (%)		GM types of VBM	MRI strength	Smoothing FWHM	Medication
	Pat	Con	Pat	Con	Pat	Con	
(17)	51	59	22.29 (3.95)	23.2 (2.58)	66.7	64.4	GM volume	3 T	8 mm	N^b^
(6)	31	37	38.0 (11.24)	39.36 (9.97)	51.6	45.9	GM volume	3 T	8 mm	Y
(12)	155	122	26.91 (5.6)	27.5 (8.2)	80.65	50	GM density	1.5 T	4 mm	Y
(7)	30	30	22.63 (3.76)	22.77 (3.34)	56.7	60.0	GM density	3 T	6 mm	Y
(13)	9	34	33.8 (13.1)	39.3 (9.5)	66.7	70.6	GM volume	1.5 T	8 mm	Y
(15)^a^	10	10	22.0 (8.2)	23.0 (7.9)	50	50	GM density	3 T	8 mm	N
(15)^a^	10	10	21.2 (7.5)	23.0 (7.9)	50	50	GM density	3 T	8 mm	N
(14)	169	212	36.39 (9.46)	33.31 (9.86)	78.2	48.6	GM volume	1.5 T	10 mm	Y
(16)	26	49	36.85 (13.7)	35.27 (11.1)	50	46.9	GM density	1.5 T	12 mm	Y

*(1) ^a^Two independent VBM comparisons between patients and healthy controls were conducted by Lui et al*.

*(2) ^b^N, patients without medication; Y, patients medicated with antipsychotics*.

*(3) Pat, patients; Con, controls; SD, standard deviation; VBM, voxel-based morphometry; GM, gray matter; FWHM, full-width at half-maximum*.

**Table 2:** **Demographic characteristics of the studies comparing relatives of patients with schizophrenia and healthy controls**.

Study	Sample size		Age in years(SD)		Male in proportions (%)		Information of relatives	GM types of VBM	MRI strength	Smoothing FWHM
	Rel	Con	Rel	Con	Rel	Con	
(17)	45	59	22.6 (3.92)	23.2 (2.58)	64.4	64.4	First-degree (siblings)	GM volume	3 T	8 mm
(6)	29	37	40.38 (15.84)	39.36 (9.97)	48.3	45.9	First-degree (parents and siblings)	GM volume	3 T	8 mm
(12)	186	122	27.5 (6.8)	27.5 (8.2)	45.7	50	First-degree (siblings)	GM density	1.5 T	4 mm
(7)	55	29	50.31 (5.1)	51.79 (5.58)	49.1	48.3	First-degree (parents)	GM density	3 T	6 mm
(13)	9	34	33.8 (13.1)	39.3 (9.5)	66.7	70.6	First-degree (MZ)	GM volume	1.5 T	8 mm
(15)^a^	10	10	41.4 (3.7)	43.2 (6.3)	37.5	40	First-degree (parents)	GM density	3 T	8 mm
(15)^a^	10	10	45.6 (6.2)	43.2 (6.3)	41.7	40	First-degree (parents)	GM density	3 T	8 mm
(14)	213	212	36.5 (9.75)	33.31 (9.86)	41.8	48.6	First-degree (siblings)	GM volume	1.5 T	10 mm
(16)	24	49	38.92 (12.9)	35.27 (11.1)	45.8	46.9	First- and second-degree	GM density	1.5 T	12 mm

*(1) ^a^Two independent VBM comparisons between relatives of patients and healthy controls were conducted by Lui et al*.

*(2) SD, standard deviation; MZ, monozygotic twins; VBM, voxel-based morphometry; GM, gray matter; FWHM, full-width at half-maximum*.

### The results of ES-SDM analysis

**Figure 2:**
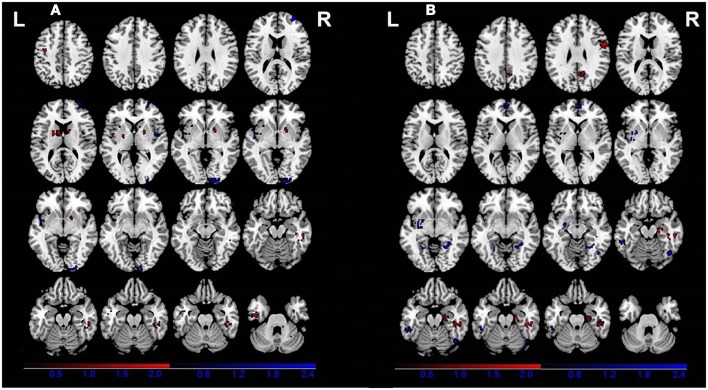
**Gray matter differences between patients (A) and relatives of patients (B) compared with healthy controls**. Areas with increased gray matter relative to controls are displayed in red, and areas with decreased gray matter are displayed in blue.

All of the nine VBM comparisons showed GM changes in patients relative to the healthy controls. Compared to the healthy controls, patients showed decreased GM in the right cuneus, the right superior frontal gyrus, the right insula, and the left *claustrum* and increased GM in the bilateral *putamen*, the right parahippocampal gyrus, the left precentral gyrus, the left inferior temporal gyrus, and the right cerebellar tonsil (Figure 2A; Table 3).

**Table 3:** **Gray matter differences between patients and healthy controls**.

Regions	Maximum		Cluster		Cluster breakdown	Jack-knife sensitivity
	Talairach coordinates	*Z* value	Uncorrected *P*	No. of voxels		No. of analyses survived by the clusters
**PATIENTS > CONTROLS**						
Right basal ganglia (Right lentiform nucleus, putamen)	20, 2, 4	2.287	0.000064226	85	Right putamen	9 out of 9
					Right caudate body	
					Right caudate head	
					Right lateral globus pallidus	
Left basal ganglia (Left lentiform nucleus, putamen)	−22, −2, 8	2.283	0.000064226	77	Left putamen	8 out of 9
					Left caudate body	
					Left lateral globus pallidus	
Right parahippocampal gyrus	38, −26, −16	2.140	0.000166988	43	Right parahippocampal gyrus	8 out of 9
					Right inferior temporal gyrus	
					Right fusiform gyrus	
Left precentral gyrus	−38, −8, 46	2.055	0.000616570	21	Left precentral gyrus	8 out of 9
					Left middle frontal gyrus	
Left inferior temporal gyrus(Left fusiform gyrus)**	−46, −12, −30	2.053	0.000616570	36	Left inferior temporal gyrus	8 out of 9
					Left fusiform gyrus	
Right cerebellar tonsil	22, −54, −34	1.831	0.002581888	13	Right cerebellar tonsil	7 out of 9
**PATIENTS < CONTROLS**						
Right cuneus	22, −98, −2	−3.877	0.000321130	90	Right cuneus	8 out of 9
					Right lingual gyrus	
					Right middle occipital gyrus	
					Right inferior occipital gyrus	
Right superior frontal gyrus	28, 60, 4	−3.645	0.000552344	78	Right superior frontal gyrus	7 out of 9
					Right middle frontal gyrus	
Right insula	42, 2, 12	−3.179	0.001451509	40	Right insula	8 out of 9
					Right superior temporal gyrus	
					Right precentral gyrus	
Left basal ganglia (Left clastrum)**	−30, 16, 2	−3.132	0.001592807	49	Left clastrum	7 out of 9
					Left superior temporal gyrus	
					Left insula	
					Left extra-nuclear	

**Shared GM alterations in patients and their unaffected relatives.

Four of the nine VBM comparisons showed no significant GM changes in unaffected relatives of patients compared to healthy controls (12, 13, 15, 16). The studies that showed significant GM changes were comparable to the studies showing negative results in terms of mean sample size of unaffected relatives [70.0 ± 81.89 vs. 57.75 ± 85.75 (*P* = 0.836)] and proportion of males [46.29 vs. 46.32% (*P* = 0.993)], but not for the mean age of the unaffected relatives (37.32 ± 7.50 vs. 29.87 ± 5.16), which was significantly different (*P* < 0.001). The results of the ES-SDM analysis from the nine VBM comparisons are shown in Table 4 and Figure 2B. Compared to healthy controls, unaffected relatives demonstrated decreased GM in the left *claustrum*, the bilateral parahippocampal gyri, the left fusiform gyrus, the right inferior temporal gyrus, and the bilateral medial prefrontal cortices and increased GM in the right hippocampus, the right fusiform gyrus, the right precentral gyrus, and the right precuneus (Figure 2B).

**Table 4:** **Gray matter differences between relatives of patients with schizophrenia and healthy controls**.

Regions	Maximum		Cluster		Cluster breakdown	Jack-knife sensitivity
	Talairach coordinates	*Z* value	Uncorrected *P*	No. of voxels		No. of analyses survived by the clusters
**RELATIVES > CONTROLS**						
Right hippocampus	24, −20, −20	2.219	0.000051381	59	Right parahippocampus	8 out of 9
					Right hippocampus	
Right fusiform gyrus**	44, −30, −20	2.200	0.000077071	71	Right fusiform gyrus	8 out of 9
					Right inferior temporal gyrus	
					Right parahippocampus	
Right precentral gyrus	56, 2, 28	2.168	0.000089917	135	Right precentral gyrus	8 out of 9
					Right inferior temporal gyrus	
					Right middle temporal gyrus	
Right precuneus	10, −60, 32	1.902	0.000924855	73	Right precuneus	8 out of 9
					Right cingulate gyrus	
**RELATIVES < CONTROLS**						
Left basal ganglia (claustrum)**	−34, −4, −6	−2.549	∼0	81	Left lentiform nucleus, putamen	8 out of 9
					Left claustrum	
					Left parahippocampal gyrus	
					Left insula	
Right parahippocampal gyrus	26, −48, −4	−2.405	0.000051381	77	Right parahippocampal gyrus	8 out of 9
					Right fusiform nucleus	
					Right declive	
					Right culmen	
Left fusiform gyrus	−52, −42, −22	−2.144	0.000488118	52	Left fusiform gyrus	8 out of 9
					Left inferior temporal gyrus	
					Left tuber	
Bilateral medial prefrontal gyri	2, 58, 2	−2.029	0.000873475	51	Right medial frontal gyrus	7 out of 9
					Left medial frontal gyrus	
Right inferior temporal gyrus	42, −64, −16	−1.950	0.001207405	53	Right declive	8 out of 9
					Right fusiform gyrus	
Left parahippocampal gyrus	−26, −54, −6	−1.919	0.001374438	16	Left parahippocampal gyrus	8 out of 9
					Left fusiform gyrus	

**Shared GM alterations in patients and their unaffected relatives.

## Discussion

Using ES-SDM meta-analysis of VBM studies in schizophrenia patients and their unaffected relatives, the current study confirmed that schizophrenia patients and their unaffected relatives share brain abnormalities, including reduced GM in the left *claustrum* and increased GM in the right fusiform gyrus. However, the patients also showed other deficits, including decreased GM in the right cuneus, the right superior frontal gyrus, and the right insula, as well as increased GM in the bilateral *putamen*, the right parahippocampal gyrus, the left precentral gyrus, the left inferior temporal gyrus, and the right cerebellar tonsil (Table 3). Their unaffected relatives also demonstrated reduced GM in other regions including the bilateral parahippocampal gyri, the left fusiform gyrus, the right inferior temporal gyri, and the medial prefrontal cortices and increased GM in the right hippocampus, the right precentral gyrus, and the right precuneus (Table 4). Thus, the current study provides evidence to support the hypothesis that GM changes in schizophrenia patients and their unaffected relatives are largely different, but share some subtle overlap.

One of the most interesting findings of the present meta-analysis is that both schizophrenia patients and their unaffected relatives shared GM changes in the left *claustrum* and the right fusiform gyrus, suggesting subtle genetic anatomical brain deficits in schizophrenia. In fact, the claustrum *belongs to the basal ganglia which* plays a critical role in the cortical-subcortical model for the pathogenesis of schizophrenia, in which prefrontal cortex dysfunction leads to disinhibited dopaminergic activity, enhanced subcortical dopamine neurotransmission in the basal ganglia, and psychosis (23, 24). The shared deficits of the basal ganglia in both patients and relatives suggest a genetic structural trait of schizophrenia, possibly reflecting endogenous overactivity of the striatal dopamine system (25). Thus, the deficits of the basal ganglia may be a core feature and a risk factor for the schizophrenia. (26) However, another possible reason for the increase GM volume of the basal ganglia in schizophrenia patients was related to the antipsychotic treatment which was reported to be the target of antipsychotic medication and found increased volume after treatment (27). The increased GM volume in the fusiform gyrus in both patients and relatives has seldom been reported and may represent a protective or compensatory phenomenon. The function of the right fusiform gyrus have been implicated in face-processing tasks requiring perception of faces and objects (28). In fact, the functional deficits of the right fusiform gyrus were also reported in both schizophrenia patients and their siblings, and these abnormalities could reflect reduced language-related lateralization in individuals with schizophrenia and their high-risk siblings (29). Another previous study indicated that loss of fusiform gyrus volume was negatively correlated with improvement in neurocognitive function as well as social cognition (30). Our study provided further evidences to support the possible genetic related GM changes of the right fusiform in schizophrenia.

However, it should be noted that the shared changes of GM volume are subtle, which suggests that the anatomical correlates of genetic liability may be small. Therefore, it may be not possible to pinpoint any single factor as being by itself necessary and sufficient to cause a psychiatric disorder. Most likely, the affected patients have multiple risk factors in different combinations that vary individually from patient to patient (31). Thus, heightened genetic risk for the unaffected first-degree relatives of schizophrenia may be reflected in small changes to cognitive functioning (9), as well as in brain structural changes. Schizophrenia is thought to result from a combination of multiple factors, such as genes, environment, antipsychotics, substance abuse, and comorbidities. However, Lui et al. observed that familial parents have more neuroanatomical abnormalities than sporadic parents (15). Furthermore, brain volume differences in twins discordant for schizophrenia were more pronounced in monozygotic than in dizygotic twins, compared with healthy control twins (32–34). This evidence suggests that the genetic contribution to brain volume alterations in schizophrenia may be subtle but prominent in subjects with a high genetic load, i.e., monozygotic discordant twins or familial parents rather than healthy siblings of patients with schizophrenia. In addition, one single factor could have multiple effects. Thus, a single gene could cause different phenotypes, depending on various interactions at the gene–gene and gene-environmental levels (genetic pleiotropism) (31). This theory is supported by recent studies that reported that the same genetic variants can cause both bipolar disorder and schizophrenia (35, 36). Furthermore, the shared GM in the *left claustrum* could be regarded with suspicion as this deep GM area is rather delicate and thus might be prone to image-registration errors.

It should also be noted that the most common deficits in GM volume in the patient group involved the cortical-striatal regions, which is consistent with a previous meta-analysis (2). The superior frontal gyrus, which showed reduced GM volume in our study, belongs to the prefrontal cortex, which is frequently thought to be a key structure in schizophrenia. The superior frontal gyrus plays an important role in managing many executive functions, such as working memory, response inhibition, and goal-directed behaviors. These cognitive abilities are typically disturbed in schizophrenia. In line with previous volumetric studies (37), we also found decreased GM in the insula, which has been suggested to be linked to clinical features such as reality distortion (38). In addition to the decreased GM, increased GM was also revealed in the patient group in the bilateral basal ganglia, the right fusiform gyrus, the left inferior temporal gyrus, the left precentral gyrus, and the right cerebellar tonsil. These findings are not surprising, as increased GM volume has also been reported in previous studies (39–42). A recent study of the largest sample of drug-naive first-episode of schizophrenia patients also revealed wide-spread increases in GM (42). The increased GM in the basal ganglia was consistent with most of the findings in an independent study of schizophrenia patients (43, 44). However, negative findings (45) or even decreased volumes of basal ganglia (46) GM have also been reported. The increased *putamen* GM may be partly attributed to exposure to antipsychotic medication, which is supported by the results of recent studies that showed increased putamen (47, 48) in first-episode drug-naïve schizophrenics after several weeks of antipsychotic treatment. Most studies included in the present meta-analysis investigated medicated schizophrenia patients, except for two studies (15, 17). Thus, the increased GM reported here could be related to antipsychotics.

Unlike the consistently positive findings in the patient groups, nearly half of the VBM comparisons (four out of nine) did not find structural differences between unaffected relatives and healthy controls. This finding suggests that the anatomical correlates of genetic liability may be weak. However, another possible reason for the negative findings in relatives is that the moderate changes in the relatives may be missed when using VBM (38). Our results of unaffected relatives are mostly consistent with Palaniyappan et al., who performed a meta-analysis of VBM studies seeking to identify the neuroanatomical correlates of genetic liability in schizophrenia. Though the primary studies that were pooled in that meta-analysis largely overlap with the studies that we identified, there are some important differences. We did not include Job et al. (49) or Marcelis et al. (50) in the present meta-analysis, as the unaffected relatives and schizophrenia patients in those two studies came from different families; thus, the patients and unaffected relatives shared no biological relationship. Furthermore, we included two other recently published studies (6, 17). The studies that showed significant GM changes were comparable to the studies showing negative results in terms of mean sample size of unaffected relatives and proportion of males, but the mean age of unaffected relatives was different.

Unexpectedly, the present meta-analysis of unaffected relatives also displayed wide-spread GM changes throughout the whole-brain (Table 4), though many areas are different from those of the patients. The left *claustrum*, the bilateral parahippocampal gyri, the left fusiform gyrus, the right inferior temporal gyrus and the bilateral medial prefrontal cortices emerged as the most significant loci with GM reductions in unaffected relatives compared to controls. The GM reduction was qualitatively consistent with some liability literature. For example, GM decreases in the parahippocampal gyrus and the medial prefrontal gyrus have been reported in previous studies of unaffected relatives of schizophrenia patients (13, 16, 51). The parahippocampal region is considered to be a part of the limbic lobe. Together with the amygdala, the parahippocampal gyrus assumes an important role in emotional processing and goal-directed processes (52). Furthermore, a recent functional study also provided evidence that hippocampal-parahippocampal dysfunction is related to genetic risk of developing schizophrenia (53). Our meta-analysis, however, did not find a grossly decreased volume of the hippocampus, which is has been reported as a pronounced change in non-psychotic first-degree relatives of schizophrenia patients in previous studies using ROI analysis (10). However, the conclusion of the previous meta-analysis was strongly influenced by a publication bias that most studies selectively measured morphometric changes in the hippocampus. Thus, the discrepancy may partly result from the different methods. VBM provides an unbiased approach to establish the presence of regional changes in GM by surveying the whole-brain.

The regions that displayed GM increases, the right parahippocampus, the right fusiform gyrus, the right precentral gyrus, and the right precuneus, have been reported in previous studies. Increased volume of the bilateral parahippocampal gyrus and the temporal lobe (though middle temporal) have been reported in unaffected relatives of patients with schizophrenia (51, 54). Furthermore, Seidman et al. (55) found increased cerebral volume in a small group of relatives. Though the exact mechanism for these increases is still unknown, it is possible that they may represent a protective or compensatory mechanism in unaffected family members, although this hypothesis is highly speculative (14). To better understand brain structure changes between patients with schizophrenia and their unaffected relatives, substantial effort is needed to search for biomarkers associated with robust genetic liability.

We acknowledge several limitations of the current meta-analysis. First, the small sample size may limit the power of our analyses, in particular sub-analyses of groups at differential genetic risk for psychosis (i.e., first-degree relatives vs. twins). Another limitation is related to the VBM methodology. Although VBM provides an unbiased approach to investigate the GM differences throughout the brain, its limitations relate to the difficulty of spatially normalizing brains, the robustness of standard parametric tests, and the interpretation of the results (56). A recent study suggested that subtle surface anatomical changes may be important for the pathophysiology of schizophrenia, and these changes may be missed when using VBM (38). Furthermore, both GM density and volume were used in the included eight studies. Though the difference of density and volume were little for VBM study, we cannot exclude the possible bias caused by the approaches. Finally, most of the patients included in the present meta-analysis were medicated. Thus, it is impossible to exclude the influence of antipsychotics on brain morphology. Cross-sectional (20) study which compared genetic high-risk subjects and first-episode antipsychotic-naive schizophrenia patients or longitudinal design (57) of high-risk subjects who finally translate to psychosis will help to analysis the genetic liability to psychosis.

Altogether, the current meta-analysis shows wide-spread GM deficits, including increased and decreased GM in both schizophrenia patients and their unaffected biological relatives. However, patients and relatives only showed subtle overlap, suggesting that the anatomical correlates of genetic liability may be weak for schizophrenia. As limitations such as small sample size and medication status could affect the findings, the relationship between GM deficits and susceptibility to developing schizophrenia remains to be established. In the future, studies of antipsychotic-naïve patients with first-episode schizophrenia and their unaffected relatives may help to clarify the genetic and environmental susceptibility to schizophrenia.

## Author Contributions

Su Lui and QiYong Gong contributed to the conceptual design of the study. Yuan Xiao and Wenjing Zhang performed the data analysis and wrote the manuscript. Su Lui and Li Yao helped perform the analysis and contribute to constructive discussions.

## Conflict of Interest Statement

The authors declare that the research was conducted in the absence of any commercial or financial relationships that could be construed as a potential conflict of interest.

## References

[1] Shenton ME Whitford TJ Kubicki M Structural neuroimaging in schizophrenia from methods to insights to treatments. Dialogues Clin Neurosci (2010) 12(3):317–32 20954428 10.31887/DCNS.2010.12.3/mshenton PMC3181976

[2] Honea R Crow TJ Passingham D Mackay CE Regional deficits in brain volume in schizophrenia: a meta-analysis of voxel-based morphometry studies. Am J Psychiatry (2005) 162(12):2233–45 10.1176/appi.ajp.162.12.2233 16330585

[3] Moran ME Pol HH Gogtay N A family affair: brain abnormalities in siblings of patients with schizophrenia. Brain (2013) 136(11):3215–26 10.1093/brain/awt116 23698280 PMC3808683

[4] Peper JS Brouwer RM Boomsma DI Kahn RS Pol H Hilleke E Genetic influences on human brain structure: a review of brain imaging studies in twins. Hum Brain Mapp (2007) 28(6):464–73 10.1002/hbm.20398 17415783 PMC6871295

[5] Stein JL Medland SE Vasquez AA Hibar DP Senstad RE Winkler AM Identification of common variants associated with human hippocampal and intracranial volumes. Nat Genet (2012) 44(5):552–61 10.1038/ng.2250 22504417 PMC3635491

[6] Oertel-Knöchel V Knöchel C Matura S Rotarska-Jagiela A Magerkurth J Prvulovic D Cortical-basal ganglia imbalance in schizophrenia patients and unaffected first-degree relatives. Schizophr Res (2012) 138(2):120–7 10.1016/j.schres.2012.02.029 22464726

[7] Tian L Meng C Yan H Zhao Q Liu Q Yan J Convergent evidence from multimodal imaging reveals amygdala abnormalities in schizophrenic patients and their first-degree relatives. PLoS One (2011) 6(12):e28794 10.1371/journal.pone.0028794 22174900 PMC3234284

[8] Bromet EJ Fennig S Epidemiology and natural history of schizophrenia. Biol Psychiatry (1999) 46(7):871–81 10.1016/S0006-3223(99)00153-5 10509170

[9] MacDonald AW Schulz SC What we know: findings that every theory of schizophrenia should explain. Schizophr Bull (2009) 35(3):493–508 10.1093/schbul/sbp017 19329559 PMC2669587

[10] Boos H Aleman A Cahn W Pol HH Kahn RS Brain volumes in relatives of patients with schizophrenia: a meta-analysis. Arch Gen Psychiatry (2007) 64(3):297–304 10.1001/archpsyc.64.3.297 17339518

[11] Palaniyappan L Balain V Liddle PF The neuroanatomy of psychotic diathesis: a meta-analytic review. J Psychiatr Res (2012) 46(10):1049–56 10.1016/j.jpsychires.2012.06.007 22790253

[12] Boos HB Cahn W van Haren NE Derks EM Brouwer RM Schnack HG Focal and global brain measurements in siblings of patients with schizophrenia. Schizophr Bull (2012) 38(4):814–25 10.1093/schbul/sbq147 21242319 PMC3406520

[13] Borgwardt SJ Picchioni MM Ettinger U Toulopoulou T Murray R McGuire PK Regional gray matter volume in monozygotic twins concordant and discordant for schizophrenia. Biol Psychiatry (2010) 67(10):956–64 10.1016/j.biopsych.2009.10.026 20006324

[14] Honea RA Meyer-Lindenberg A Hobbs KB Pezawas L Mattay VS Egan MF Is gray matter volume an intermediate phenotype for schizophrenia? A voxel-based morphometry study of patients with schizophrenia and their healthy siblings. Biol Psychiatry (2008) 63(5):465–74 10.1016/j.biopsych.2007.05.027 17689500 PMC2390785

[15] Lui S Deng W Huang X Jiang L Ouyang L Borgwardt SJ Neuroanatomical differences between familial and sporadic schizophrenia and their parents: an optimized voxel-based morphometry study. Psychiatry Res (2009) 171(2):71–81 10.1016/j.pscychresns.2008.02.004 19168334

[16] McIntosh AM Job DE Moorhead TWJ Harrison LK Forrester K Lawrie SM Voxel-based morphometry of patients with schizophrenia or bipolar disorder and their unaffected relatives. Biol Psychiatry (2004) 56(8):544–52 10.1016/j.biopsych.2004.07.020 15476683

[17] Hu M Li J Eyler L Guo X Wei Q Tang J Decreased left middle temporal gyrus volume in antipsychotic drug-naive, first-episode schizophrenia patients and their healthy unaffected siblings. Schizophr Res (2013) 144(1):37–42 10.1016/j.schres.2012.12.018 23360727

[18] Stroup DF Berlin JA Morton SC Olkin I Williamson GD Rennie D Meta-analysis of observational studies in epidemiology. JAMA (2000) 283(15):2008–12 10.1001/jama.283.15.2008 10789670

[19] Radua J Mataix-Cols D Voxel-wise meta-analysis of grey matter changes in obsessive-compulsive disorder. Br J Psychiatry (2009) 195(5):393–402 10.1192/bjp.bp.108.055046 19880927

[20] Radua J Mataix-Cols D Phillips M El-Hage W Kronhaus D Cardoner N A new meta-analytic method for neuroimaging studies that combines reported peak coordinates and statistical parametric maps. Eur Psychiatry (2012) 27(8):605–11 10.1016/j.eurpsy.2011.04.001 21658917

[21] Turkeltaub PE Eden GF Jones KM Zeffiro TA Meta-analysis of the functional neuroanatomy of single-word reading: method and validation. Neuroimage (2002) 16(3):765–80 10.1006/nimg.2002.1131 12169260

[22] Wager TD Lindquist M Kaplan L Meta-analysis of functional neuroimaging data: current and future directions. Soc Cogn Affect Neurosci (2007) 2(2):150–8 10.1093/scan/nsm015 18985131 PMC2555451

[23] Weinberger DR Implications of normal brain development for the pathogenesis of schizophrenia. Arch Gen Psychiatry (1987) 44(7):660–9 10.1001/archpsyc.1987.01800190080012 3606332

[24] Yoon JH Minzenberg MJ Raouf S D’Esposito M Carter CS Impaired prefrontal-basal ganglia functional connectivity and substantia nigra hyperactivity in schizophrenia. Biol Psychiatry (2013) 74(2):122–9 10.1016/j.biopsych.2012.11.018 23290498 PMC3620727

[25] Howes OD Kapur S The dopamine hypothesis of schizophrenia: version III – the final common pathway. Schizophr Bull (2009) 35(3):549–62 10.1093/schbul/sbp006 19325164 PMC2669582

[26] Perez-Costas E Melendez-Ferro M Roberts RC Basal ganglia pathology in schizophrenia: dopamine connections and anomalies. J Neurochem (2010) 113(2):287–302 10.1111/j.1471-4159.2010.06604.x 20089137 PMC2929831

[27] Smieskova R Fusar-Poli P Allen P Bendfeldt K Stieglitz R Drewe J The effects of antipsychotics on the brain: what have we learnt from structural imaging of schizophrenia? A systematic review. Curr Pharm Des (2009) 15(22):2535–49 10.2174/138161209788957456 19689326

[28] Gur RE Calkins ME Gur RC Horan WP Nuechterlein KH Seidman LJ The consortium on the genetics of schizophrenia: neurocognitive endophenotypes. Schizophr Bull (2007) 33(1):49–68 10.1093/schbul/sbl055 17101692 PMC2632287

[29] MacDonald AW Thermenos HW Barch DM Seidman LJ Imaging genetic liability to schizophrenia: systematic review of FMRI studies of patients’ nonpsychotic relatives. Schizophr Bull (2009) 35(6):1142–62 10.1093/schbul/sbn053 18556667 PMC2762618

[30] Eack SM Hogarty GE Cho RY Prasad KM Greenwald DP Hogarty SS Neuroprotective effects of cognitive enhancement therapy against gray matter loss in early schizophrenia: results from a 2-year randomized controlled trial. Arch Gen Psychiatry (2010) 67(7):674–82 10.1001/archgenpsychiatry.2010.63 20439824 PMC3741671

[31] Licinio J Wong M A novel conceptual framework for psychiatry: vertically and horizontally integrated approaches to redundancy and pleiotropism that co-exist with a classification of symptom clusters based on DSM-5. Mol Psychiatry (2013) 18(8):846–8 10.1038/mp.2013.90 23896674

[32] Baaré WF van Oel CJ Hulshoff Pol HE Schnack HG Durston S Sitskoorn MM Volumes of brain structures in twins discordant for schizophrenia. Arch Gen Psychiatry (2001) 58(1):33–40 10.1001/archpsyc.58.1.33 11146756

[33] Hulshoff Pol HE Brans RG van Haren NE Schnack HG Langen M Baaré WF Gray and white matter volume abnormalities in monozygotic and same-gender dizygotic twins discordant for schizophrenia. Biol Psychiatry (2004) 55(2):126–30 10.1016/S0006-3223(03)00728-5 14732591

[34] Hulshoff Pol HE Schnack HG Mandl RC Brans RG van Haren NE Baaré WF Gray and white matter density changes in monozygotic and same-sex dizygotic twins discordant for schizophrenia using voxel-based morphometry. Neuroimage (2006) 31(2):482–8 10.1016/j.neuroimage.2005.12.056 16497519

[35] Dwyer S Williams H Jones I Jones L Walters J Craddock N Investigation of rare non-synonymous variants at ABCA13 in schizophrenia and bipolar disorder. Mol Psychiatry (2011) 16(8):790–1 10.1038/mp.2011.2 21283083

[36] Williams HJ Norton N Dwyer S Moskvina V Nikolov I Carroll L Fine mapping of ZNF804A and genome-wide significant evidence for its involvement in schizophrenia and bipolar disorder. Mol Psychiatry (2010) 16(4):429–41 10.1038/mp.2010.36 20368704 PMC3918934

[37] Makris N Goldstein JM Kennedy D Hodge SM Caviness VS Faraone SV Decreased volume of left and total anterior insular lobule in schizophrenia. Schizophr Res (2006) 83(2):155–71 10.1016/j.schres.2005.11.020 16448806

[38] Palaniyappan L Liddle PF Differential effects of surface area, gyrification and cortical thickness on voxel based morphometric deficits in schizophrenia. Neuroimage (2012) 60(1):693–9 10.1016/j.neuroimage.2011.12.058 22227049

[39] Ellison-Wright I Bullmore E Anatomy of bipolar disorder and schizophrenia: a meta-analysis. Schizophr Res (2010) 117(1):1–12 10.1016/j.schres.2009.12.022 20071149

[40] Glahn DC Laird AR Ellison-Wright I Thelen SM Robinson JL Lancaster JL Meta-analysis of gray matter anomalies in schizophrenia: application of anatomic likelihood estimation and network analysis. Biol Psychiatry (2008) 64(9):774–81 10.1016/j.biopsych.2008.03.031 18486104 PMC5441233

[41] Kawasaki Y Suzuki M Nohara S Hagino H Takahashi T Matsui M Structural brain differences in patients with schizophrenia and schizotypal disorder demonstrated by voxel-based morphometry. Eur Arch Psychiatry Clin Neurosci (2004) 254(6):406–14 10.1007/s00406-004-0522-1 15538599

[42] Ren W Lui S Deng W Li F Li M Huang X Anatomical and functional brain abnormalities in drug-naive first-episode schizophrenia. Am J Psychiatry (2013) 170(11):1308–16 10.1176/appi.ajp.2013.12091148 23732942

[43] Staal WG Pol HEH Schnack HG Hoogendoorn ML Kahn R Structural brain abnormalities in patients with schizophrenia and their healthy siblings. Am J Psychiatry (2000) 157(3):416–21 10.1176/appi.ajp.157.3.416 10698818

[44] Mamah D Wang L Barch D de Erausquin GA Gado M Csernansky JG Structural analysis of the basal ganglia in schizophrenia. Schizophr Res (2007) 89(1):59–71 10.1016/j.schres.2006.08.031 17071057 PMC1839817

[45] Gunduz H Wu H Ashtari M Bogerts B Crandall D Robinson DG Basal ganglia volumes in first-episode schizophrenia and healthy comparison subjects. Biol Psychiatry (2002) 51(10):801–8 10.1016/S0006-3223(01)01345-2 12007454

[46] Westmoreland Corson P Nopoulos P Andreasen NC Heckel D Arndt S Caudate size in first-episode neuroleptic-naive schizophrenic patients measured using an artificial neural network. Biol Psychiatry (1999) 46(5):712–20 10.1016/S0006-3223(99)00079-7 10472424

[47] Glenthoj A Glenthoj BY Mackeprang T Pagsberg AK Hemmingsen RP Jernigan TL Basal ganglia volumes in drug-naive first-episode schizophrenia patients before and after short-term treatment with either a typical or an atypical antipsychotic drug. Psychiatry Res (2007) 154(3):199–208 10.1016/j.pscychresns.2006.10.002 17360162

[48] Li M Chen Z Deng W He Z Wang Q Jiang L Volume increases in putamen associated with positive symptom reduction in previously drug-naive schizophrenia after 6 weeks antipsychotic treatment. Psychol Med (2012) 42(07):1475–83 10.1017/S0033291711002157 22030695

[49] Job DE Whalley HC McConnell S Glabus M Johnstone EC Lawrie SM Voxel-based morphometry of grey matter densities in subjects at high risk of schizophrenia. Schizophr Res (2003) 64(1):1–13 10.1016/S0920-9964(03)00158-0 14511796

[50] Marcelis M Suckling J Woodruff P Hofman P Bullmore E van Os J Searching for a structural endophenotype in psychosis using computational morphometry. Psychiatry Res (2003) 122(3):153–67 10.1016/S0925-4927(02)00125-7 12694890

[51] Goghari VM Rehm K Carter CS MacDonald AW Regionally specific cortical thinning and gray matter abnormalities in the healthy relatives of schizophrenia patients. Cereb Cortex (2007) 17(2):415–24 10.1093/cercor/bhj158 16547347

[52] Laurens KR Kiehl KA Liddle PF A supramodal limbic-paralimbic-neocortical network supports goal-directed stimulus processing. Hum Brain Mapp (2005) 24(1):35–49 10.1002/hbm.20062 15593271 PMC6871708

[53] Di Giorgio A Gelao B Caforio G Romano R Andriola I D’Ambrosio E Evidence that hippocampal-parahippocampal dysfunction is related to genetic risk for schizophrenia. Psychol Med (2012) 1(1):1–11 10.1017/S0033291712002413 23111173

[54] Seidman LJ Pantelis C Keshavan MS Faraone SV Goldstein JM Horton NJ A review and new report of medial temporal lobe dysfunction as a vulnerability indicator for schizophrenia: a magnetic resonance imaging morphometric family study of the parahippocampal gyrus. Schizophr Bull (2003) 29(4):803–30 10.1093/oxfordjournals.schbul.a007048 14989416

[55] Seidman LJ Faraone SV Goldstein JM Goodman JM Kremen WS Matsuda G Reduced subcortical brain volumes in nonpsychotic siblings of schizophrenic patients: a pilot magnetic resonance imaging study. Am J Med Genet (1997) 74(5):507–14 10.1002/(SICI)1096-8628(19970919)74:5<507::AID-AJMG11>3.0.CO;2-G 9342202

[56] Mechelli A Price CJ Friston KJ Ashburner J Voxel-based morphometry of the human brain: methods and applications. Curr Med Imaging Rev (2005) 1(2):105–13 10.2174/1573405054038726

[57] McIntosh AM Owens DC Moorhead WJ Whalley HC Stanfield AC Hall J Longitudinal volume reductions in people at high genetic risk of schizophrenia as they develop psychosis. Biol Psychiatry (2011) 69(10):953–8 10.1016/j.biopsych.2010.11.003 21168123

